# Activation of the mTOR/ Akt pathway in thymic epithelial cells derived from thymomas

**DOI:** 10.1371/journal.pone.0197655

**Published:** 2019-03-21

**Authors:** Jean-Michel Maury, Claire Merveilleux du Vignaux, Gabrielle Drevet, Virginie Zarza, Lara Chalabreysse, Carine Maisse, Barbara Gineys, Christine Dolmazon, François Tronc, Nicolas Girard, Caroline Leroux

**Affiliations:** 1 IVPC UMR754 INRA, Univ Lyon, Université Claude Bernard Lyon 1, EPHE, Lyon, France; 2 Department of Thoracic Surgery, Lung and Heart-lung Transplantation, Groupement Hospitalier Est, HCL, Lyon, France; 3 National Expert Center for Thymic Malignancies, Réseau Tumeurs THYMiques et Cancer (RYTHMIC), Lyon, France; 4 Department of Respiratory Diseases, Groupement Hospitalier Est, HCL Lyon, France; 5 Department of Pathology, Groupement Hospitalier Est, HCL, Lyon, France; 6 Institut du Thorax Curie Montsouris, Institut Curie, Paris, France; Univerzitet u Beogradu, SERBIA

## Abstract

The pathogenesis of thymic epithelial tumors remains poorly elucidated. The PIK3/Akt/mTOR pathway plays a key role in various cancers; interestingly, several phase I/II studies have reported a positive effect of mTOR inhibitors in disease control in thymoma patients. A major limit for deciphering cellular and molecular events leading to the transformation of thymic epithelial cells or for testing drug candidates is the lack of reliable *in vitro* cell system. We analyzed protein expression and activation of key players of the Akt/ mTOR pathway namely Akt, mTOR, and P70S6K in eleven A, B and AB thymomas as well as in normal thymuses. While only Akt and phospho-Akt were expressed in normal thymuses, both Akt and mTOR were activated in thymomas. Phospho-P70S6K was expressed in all thymic tumors whatever their subtypes, and absent in normal thymus. Interestingly, we report the activation of Akt, mTOR and P70S6 proteins in primary thymic epithelial cells maintained for short period of time after their derivation from seven AB and B thymomas. Finally, we showed that rapamycin (100 nM) significantly reduced proliferation of thymoma- derived epithelial cells without inducing cell death. Our results suggest that the activation of the Akt/ mTOR pathway might participate to the cell proliferation associated with tumor growth. Ultimately, our data enhance the potential role of thymic epithelial cells derived from tissue specimens for *in vitro* exploration of molecular abnormalities in rare thymic tumors.

## Introduction

Thymic epithelial tumors (TETs) are rare epithelial malignancies (0.2–1.5%) of the anterior mediastinum, with an estimated incidence of about 1.3–3.2 cases per million worldwide [1]. The WHO classification distinguishes thymomas and thymic carcinomas [2]. Thymomas are defined as A, AB, B1, B2, B3 sub-types according to the morphology of tumor epithelial cells, the proportion of non- tumoral thymic lymphocytes (decreasing from B1 to B3) that are associated with tumor cells, and their similarities to normal thymic architecture. Thymic carcinomas present with a high degree of epithelial cells atypia associated with a loss of normal thymic architecture.

Surgical resection is the corner stone of the multimodal treatment of thymomas [3]. Tumor stage [4] and radical complete surgical resection have been shown as independent prognosis factor of best outcome [5–7]. Advanced or metastatic cases are treated with induction chemotherapy, surgery, combined radiation-chemotherapy [8–11] with variable outcomes [12–15]. Meanwhile about 30% of patients are presenting with recurrences requiring systemic treatment.

The pathogenesis of thymic epithelial tumors remains poorly elucidated. Sustained efforts have been made to characterize molecular abnormalities occurring in TETs to improve their treatment and eventually the patient prognosis. Sequencing of 197 cancer-related genes revealed the presence of non-synonymous somatic mutations in over 60% thymic carcinomas and barely 15% thymomas [16]. The most frequent mutations (26% of thymic carcinomas) were located in the p53 tumor suppressor gene [17]. The Cancer Genome Atlas recently reported results using multi-platform omics analyses on 117 TETs, leading to identify four subtypes accordingly to their genomic hallmarks [17]. GTF2I was confirmed as an oncogene associated with type A thymoma, and mutations in HRAS, NRAS, and TP53 were identified in thymomas. A major limit of those studies was the use of tumor tissue specimens precluding specific analysis of epithelial tumor cells while lymphocytes may present with a high level of expression of genes related to carcinogenesis [17–23]

The PIK3/ Akt/ mTOR pathway plays a key role in various cancers and among them thymic tumors. Mutations of genes encoding regulatory subunit of PIK3 have been reported in a tumorigenic thymic carcinoma cell line, using targeted exome sequencing, predicting the efficacy of PIK3 inhibitors [24]. Several phase I/ II studies of mTOR inhibitors were reported in advanced thymic epithelial tumors, reporting on high disease control rates [25–27]. Meanwhile the cellular dysregulation of the Akt/ mTOR pathway has not been described in thymomas. Using primary thymic epithelial cells derived from A, AB and B thymomas, we report the dysregulation of the Akt/ mTOR pathway in thymomas and the anti-proliferative effect of rapamycin on thymic epithelial cells.

## Materials and methods

### Biological samples

Between January 2015 and December 2017, thymic samples from patients that have undergone removal surgery for thymic tumors (N = 12) (Table 1) or cardiac surgery (N = 2) for normal thymuses have been included. Tumoral and normal tissues and their associated data (Table 1) were obtained from the CardioBiotec biobank (CRB-HCL, Hospices Civils de Lyon BB-0033-0046), a center for biological resources authorized by the French ministry of social affairs and health. Patients with thymic epithelial tumors were identified by the departments of pulmonary medicine and thoracic oncology, and thoracic surgery (Groupement Hospitalier Est, HCL, Lyon). All samples were collected and used in accordance with the ethical rules of the biobank and in agreement with the French legislation. All patients signed a written informed consent. Immediately after surgery, the thymic tumors were placed in RPMI 1640 cell medium supplemented with penicillin and streptomycin and processed in a biosafety level 2 lab in the next two hours for cell derivation or snap frozen in liquid nitrogen for further use.

**Table 1:** Clinical features of thymomas included in the study.

Patient #	Age (yr)	Gender	Myasthenia gravis	Tumor size (cm)	Previous chemotherapy	Subtype^1^	Staging^2^	TNM^3^
3153	73	M	No	?^4^	No	A (pleural metastasis)	IVA	T3N0M1a
3154	70	M	No	5.7	No	A (Micronodular with lymphoid stroma)	IIA	T1aN0M0
2635	57	M	No	6	No	AB	I	T1aN0M0
2646	83	F	No	5	No	AB	I	T1aN0M0
2836	46	M	No	10	No	AB	I	T1aN0M0
3147	70	M	No	11	No	AB	III	T2N0M0
3148	65	F	No	3.5	Yes^5^	AB	I	T1aN0M0
3152	68	F	No	3.5	No	AB	I	T1aN0M0
3146	71	M	Yes	3.2	No	B2	IIA	T1aN0M0
3149	63	F	No	9	Yes^6^	B2/ B3	I	T1aN0M0
3150	66	M	Yes	6	No	B2/ B3	III	T3N0M0
2637	68	M	No	7	No	Thymic carcinoma	III	T3N0M0

^1^ Subtype according to the WHO classification

^2^ staging according to the Masaoka-Koga classification

^3^ TNM according to the ITMIG guidelines

^4^ unknown, pleural metastasis

^5^ received Caelyx therapy for Kaposi treatment

^6^ received cisplatin, adriamycin and cyclophosphamide neo-adjuvant treatment.

### Derivation of thymic epithelial cells

Thymic epithelia cells (TECs) were obtained from thymic epithelial tumors as previously described with minor modifications [28, 29]. Thymic tissues were immediately placed in ice cold RPMI 1640 medium, cut in 1–3 mm^3^ pieces and transferred in "Liberase digestion solution" (RPMI 1640 medium supplemented with 0.5 U/ml Liberase (Roche) and 0.1% w/v DNase I) with ~ 2 ml of digestion solution/ cm^3^ of tissues. After 20 min incubation at 37°C under gentle agitation, supernatants were collected, mixed (v/v) with 1X PBS supplemented with 0.1% bovine serum albumin, and 0.5 mM EDTA, and centrifuged at 480g for 10 min at 4°C. The digestion procedure was repeated 4–6 times. Cells were counted in trypan blue using a Cellometer (Nexcelon BioSciences), seeded at 2–4.10^6^ cells/ cm^2^ in "TEC medium" (RPMI 1640 medium supplemented with 2% Ultroser serum substitute (Pall corporation) and penicillin/streptomycin) and incubated at 37°C, 5% CO_2_ in humid atmosphere. After 24 hours, cell culture supernatants were centrifuged at 480g for10 min at 4°C to eliminate non-adherent cells, supplemented with an equal volume of new "TEC medium" and added to the cultured cells. Cells were checked daily and passaged at confluence with trypsin EDTA solution.

### Phenotypic characterization of primary thymic epithelial cells

Primary thymic epithelial cells were cultured on treated glass slides (LabTek, Thermo Scientific) in "TEC medium", rinsed in PBS and fixed with ice cold acetone. After 20 min rehydration in PBS, cells were incubated for one hour at room temperature with the BM4048 mouse monoclonal anti-cytokeratin (Acris) or mouse monoclonal anti-vimentin (Sigma) antibodies diluted in PBS supplemented with 1% bovine serum albumin as recommended. After PBS washes, cells were incubated for one hour at room temperature with anti-mouse IgG Dylight antibodies (Eurobio). Nuclei were stained with DAPI for 10 min and mounted with Fluoromount G (Electron Microscopy Sciences). Microscopic examinations were performed using an Axio-Imager Z1 epifluorescence microscope and analyzed using the Zen software (Zeiss).

### Protein expression in thymic tissues and thymic epithelial cells

Proteins were prepared from thymic tissues and primary thymic epithelial cells using respectively T-PER extraction reagent or M-PER extraction reagent (Thermo Fisher) supplemented with "HALT protease and "phosphatase inhibitor cocktail” (Thermo Fisher). Lysates were homogenized by sonication on ice and proteins were quantified in the collected supernatants. Twenty to thirty micrograms of total proteins were separated on SDS-PAGE, transferred onto PVDF membrane and used for the detection of Akt with "Akt1 Precision Ab antibody" (BIORAD VMA00253), phospho-Akt with "phospho-Akt (Ser 473) antibody" (Cell signaling, 9271S), mTOR with "mTOR PrecisionAb Antibody" (BIORAD, VPA00174), phospho mTOR with "phospho-mTOR (Ser2448) Antibody" (Cell signaling, 2971), phospho p70S6k with "phospho-p70 S6 Kinase (Thr389) Antibody" (Cell signaling, 9205) and β-actin (Monoclonal Anti-β-Actin−Peroxidase antibody, Sigma) as recommended. Immunoreactive bands were detected with goat anti-rabbit IgG (whole molecule)-peroxidase antibodies produced in goat (Sigma) and revealed using the "Clarity Max Western ECL Blotting Substrate" (BIORAD) on a "ChemiDoc Imaging System" (BIORAD).

### Thymic epithelial cells proliferation upon rapamycin exposure

Primary thymic epithelial cells (5000 cells/well) derived from patients #3147 (AB type), #3146 (B2 type) and #3149 (B2/B3 type) were incubated in "TEC medium" in 96 well plates for twelve hours then treated with 1, 10 or 100 nM rapamycin (Sigma). Controls with DMSO or untreated cells have been included. Proliferation was measured 24, 48 and 72 hours after rapamycin treatment with the “CellTiter-Glo Luminescent Cell viability assay” (Promega). All tests have been repeated at least twice and performed in triplicates.

### Cell death of thymic epithelial cells upon cisplatin and rapamycin exposure

Primary thymic epithelial cells (5.10^4^ cells/well) derived from thymomas #3146 (B2), #3147 (AB) and #3149 (B2/B3) were incubated in "TEC medium" in 6 well plates for twelve hours then treated with 100 nM rapamycin (Sigma) or 10 μM cisplatin (Promega). Negative controls with untreated cells have been included. HeLa cells treated with 10 μM cisplatin have been used as positive controls of induced cell death. Cell death assay was measured by flow cytometry (Becton Dickinson) 24- and 48-hours post-treatment, using 10μg/ml propidium iodide.

### Mutations analysis in the PIK3CA, PIK3R1and GTF2I genes

Mutations of PIK3CA, PIK3R1 and GTF2i have been analyzed using primers in exons 1 to 21 for *PIK3CA* (GenBank NM06218.3), exons 5 to 16 for *PIK3R1* (GenBank NM181523) and exons 5–15 for *GTF2I* (GenBank NM001518.4) gene (Table 2).

**Table 2:** Primers used to amplify GTF2i and PIK3 genes.

Gene	Region	5'-3' sequence (5'- 3')
PIK3CA	Exons 1 to 4	FOR—AAGAGCCCCGAGCGTTTCT
		REV- TGCTTCAGCAATTACTTGTTCTGG
	Exons 4 to 11	FOR—ACCATGACTGTGTACCAGAACAA
		REV- ACACAATAGTGTCTGTGACTCCA
	Exons 18 to 21	FOR—AAGGAGAAATATATGATGCAGCCA
		REV- CCAGAGTGAGCTTTCATTTTCTCA
PIK3R1	Exons 5 to 9	FOR—ACGTTTTGGCTGACGCTTTC
		REV- GGTTAATGGGTCAGAGAAGCCA
	Exons 10 to 16	FOR—ACTCTTACACTAAGGAAAGGGGGA
		REV- GCCTCAGGGTGGCTGAACT
GTF2I	Exons 10 to 15	FOR—TGAAGGCACAGAAATGGA
		REV- ACCATTCTTCCTTTACTCC

Briefly, RNAs were extracted with the "Pure Link RNA minikit" (Ambion), reverse transcribed using the "iScript cDNA synthesis kit" (Bio-Rad) and amplified with the "KAPA HIFI hotstart polymerase" (Clinisciences) for 35 cycles (2 min at 98°C, 15 sec at 60°C and 30 sec at 72°C) on a Mic qPCR system (BioMolecularSystem). Amplicons were controlled on agarose gel, sequenced (GTAC Biotech) and analyzed with the Vector NTI software (Invitrogen). The potential impact of identified mutation was analyzed with the "PolyPhen-2 (Polymorphism Phenotyping v2) predictive model".

### Statistical analysis

Statistical analyses (threshold of α = 0.05) were performed using the t test with the "GraphPad Prism" (GraphPad) software. All tests were done with a significant threshold of α = 0.05.

## Results

### Clinical features

From January 2015 to December 2017, twelve patients (8 males and 4 females, mean age of 66.67 ± 8.99 years (46–83)) who have undergone surgery (Table 1) for thymic epithelial tumors (3154, 2635, 2646, 2836, 3147, 3148, 3152, 3146, 3149, 3150, 2637) or for pleural relapse of a type A thymoma removed 25 months before the surgery (3153) have been included in our study (Table 1).

Among them, patients 3148 and 3149 received chemotherapy before surgery to respectively treat cutaneous Kaposi lesions with Caelyx and initially locally advanced thymoma with cisplatin, adriamycin and cyclophosphamide. Two patients (3146 and 3150) presented myasthenia gravis and were treated with intra venous polyvalent immunoglobulins one week before surgery (Table 1). According to the WHO pathological classification, tumors were of type AB for 6 patients (2635, 2646, 2836, 3147, 3148, and 3152), B2 for 3 patients (3146, 3149, 3150) and thymic carcinoma for patient 2637 (Table 1). Tissues from patient 3153 and 3154 were respectively identified as pleural metastasis of subtype A thymoma and as micronodular thymoma with lymphoid stroma, a rare presentation of subtype A thymoma. According to the Masaoka classification, tumors were of stage I for six patients, IIA for two patients, III for three patients and IVA for one patient (Table 1).

### Derivation of thymic epithelial cells

**Fig 1:**
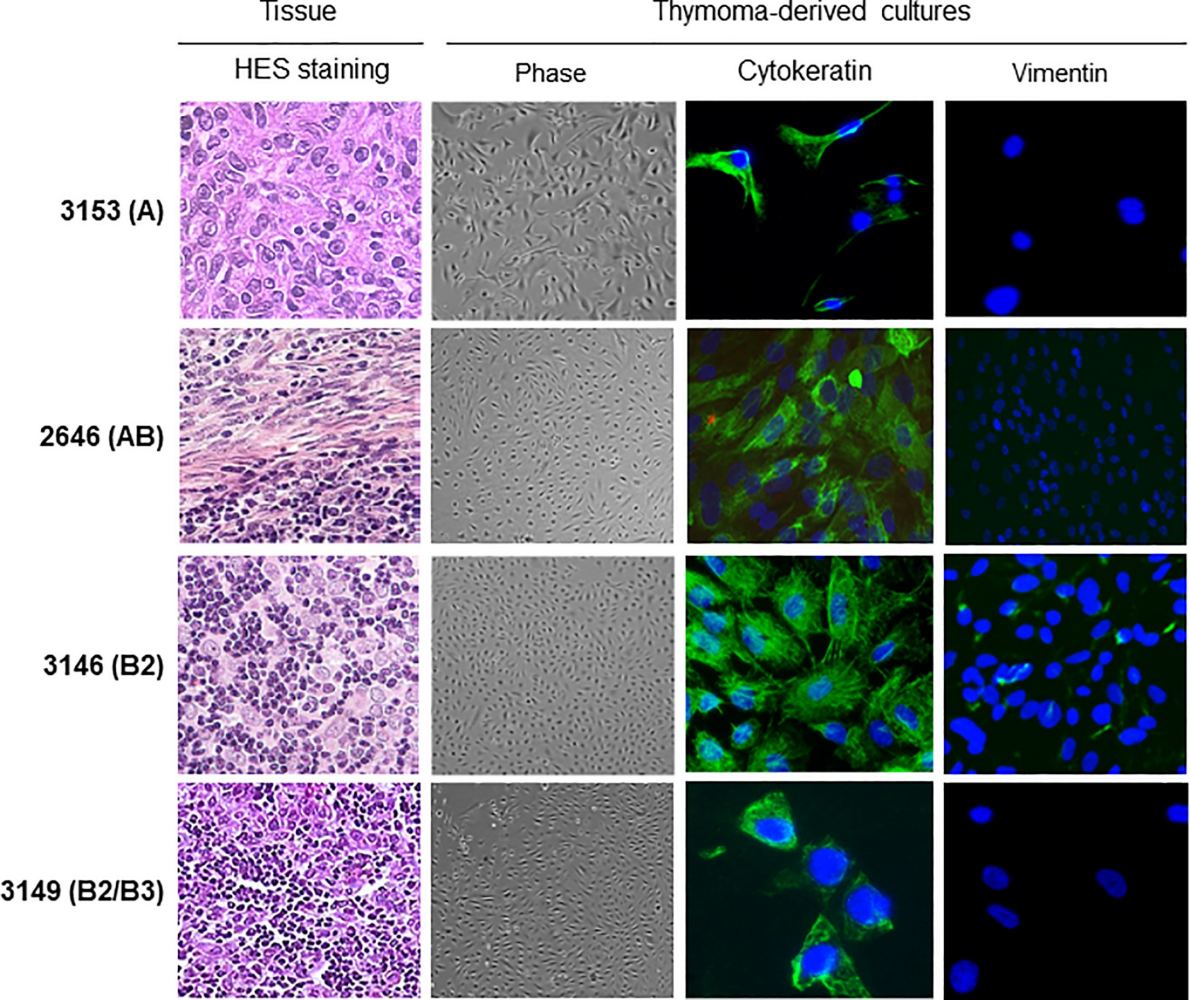
**Primary thymic epithelial cells derived from A, AB and B thymomas**. Representative cell cultures derived from patients 3153 (pleural metastasis of A thymoma), 2646 (AB thymoma), 3146 (B2 thymoma) and 3149 (B2/ B3 thymoma). HES (hematoxylin- eosin- saffron) staining were used to characterize the thymic tissues. Thymoma-derived cells were observed daily by phase microscopy and stained for their expression of cytokeratin and vimentin. Nuclei stained with DAPI (blue).

Primary thymic epithelial cells were successfully derived from the twelve tumors immediately after removal and expanded in culture. Daily observation under phase microscope showed that cells had an epithelioid morphology (Fig 1). Cells proliferated, and the epithelial morphology was maintained along the study up to passages 6 to 7, corresponding to a median time of culture of 56 days. The thymoma-derived primary thymic cells expressed cytokeratin (Fig 1), a marker of epithelial cells, with a mean of 79% positive cells except for cells derived from the thymic carcinoma 2637 that were negative for cytokeratin but expressed vimentin.

To summarize, we successfully derived primary thymic epithelial cells from various types of thymic tumors as well as pleural metastasis. These cells expressed cytokeratin and were able to proliferate *in vitro* over several passages. The subsequent analyses (detection of protein as well as proliferation studies) were performed on early passages, to be as close as possible to the *in vivo* phenotype.

### Mutations of PiK3 and GTF2i genes

**Fig 2:**
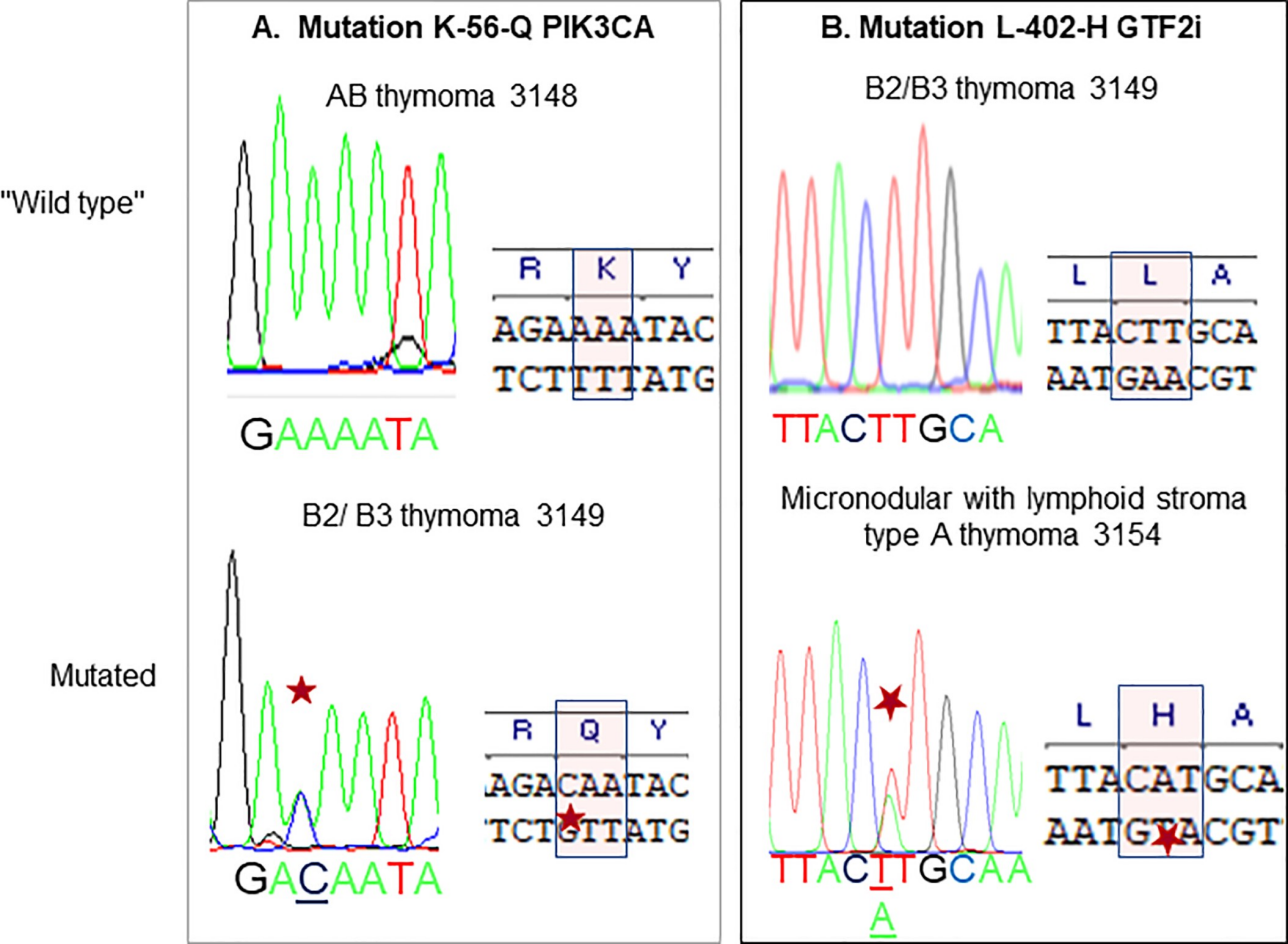
Mutations in PIK3CA and GTF2i. PIK3CA and GTF2i have been amplified from total RNA extracted from thymoma, sequenced and compared to reference sequences. A. C/A mutation in exon 2 of PIK3CA in B2/ B3 thymoma #3149. B. T/A mutation in exon 15 of GTF2i in micronodular with lymphoid stroma type A thymoma #3154.

A screening of PIK3 and GTF2I mutations was performed on all tumors. Among the 12 tumors, only patient 3149 (B2/ B3 thymoma) carried a non-conservative A/C transversion localized on position 56 of exon 2 of PIK3CA (Fig 2A). The mutation induced a K → Q amino-acid change, predicted as deleterious with PolyPhen-2.

We also detected a GTF2I mutation for tumor 3154, reported as a micronodular thymoma with lymphoid stroma, a rare presentation of type A thymoma (Fig 2B). The mutation located on exon 15 was associated with a non-conservative T/A transversion leading to L→ H amino-acid change in the deduced amino-acid sequence (Fig 2B).

### Activation of the Akt/ mTOR/ P70S6K pathway in thymomas

**Fig 3:**
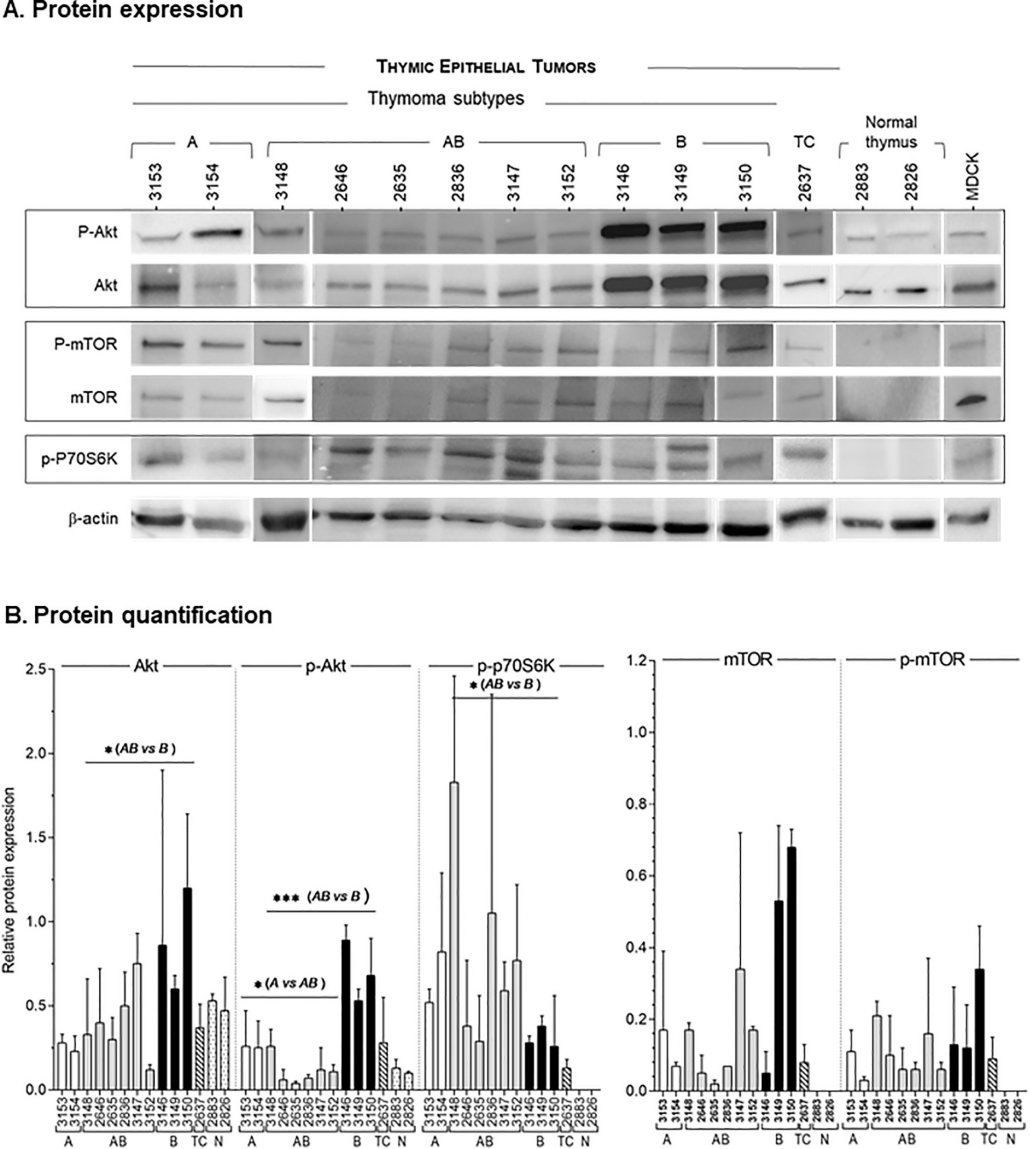
Activation of the Akt/ mTOR pathway in thymic epithelial tumors. A. Total and phosphorylated protein expression was analyzed using antibodies directed against total Akt and phosphorylated- Akt (P- Akt) (60 kDa), total mTOR and phosphorylated- mTOR (P- mTOR) (289 kDa), phosphorylated P70S6K (P- P70S6K) (70 kDa) and β-Actin (40 kDa). B. Protein expression (Akt, phospho- Akt, phospho- P70S6K, mTOR, phospho- mTOR) has been measured and expressed as [protein of interest/ actin] relative expression in A (white bars), AB (grey bars) and B (B2 and B2/ B3; black bars) thymomas, thymic carcinoma (TC, hatched bars) or normal thymus (N, dotted bars). Detection have been repeated at least 3 times. Data have been statistically analyzed with an unpaired T test.

The Akt/ mTOR pathway is a key pathway implicated in cell proliferation. We analyzed its activation in thymomas as well as in normal thymuses from cardiac surgery. Akt, mTOR and P70S6K were activated in all thymomas, as shown by the detection of phosphorylated proteins (Fig 3A). B2 thymomas expressed significantly higher levels of Akt and phospho- Akt than A or AB subtypes (Fig 3B). Total mTOR and Phospho-mTOR were expressed in thymomas, with no significant differences between subtypes but undetectable in normal thymuses. Phospho- P70S6K was absent in normal thymuses, expressed in all thymic tumors whatever their subtypes, and significantly higher in AB as compared to B thymomas.

Overall, the Akt/ mTOR pathway was activated in A, AB and B thymomas as demonstrated by the detection of phosphorylated Akt, mTOR and P70S6K proteins, with various level of expression that probably reflected the relative frequency of tumoral and non-tumoral cells within the tumors.

### Activation of the Akt/ mTOR pathway in thymic epithelial cells derived from thymomas

**Fig 4:**
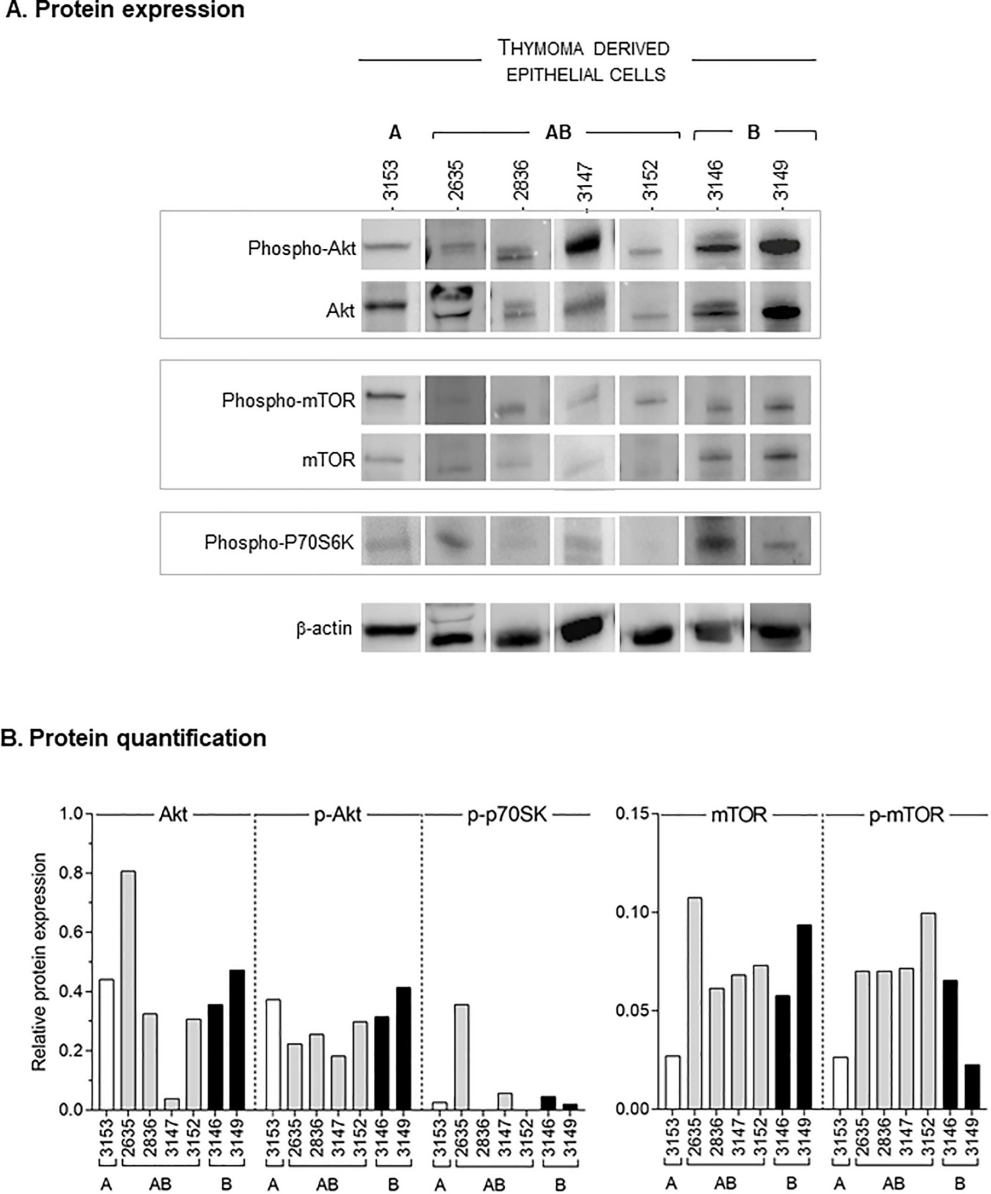
Activation of the Akt/ mTOR pathway in thymoma-derived thymic epithelial cells. **A.** Total and phosphorylated protein expression was analyzed using antibodies directed against total Akt and phosphorylated- Akt (phospho- Akt), total mTOR and phosphorylated- mTOR (phospho- mTOR), phosphorylated P70S6K (phospho- P70S6K) and β-Actin in thymic epithelia cells derived from A, AB or B2 thymomas. B. Protein expression has been measured and expressed as [protein of interest/ actin] relative expression in A (white bars), AB (grey bars) and B (B2 and B2/ B3; black bars) thymomas.

To determine the role of the Akt/ mTOR pathway in proliferation of tumoral thymic epithelial cells, we analyzed expression of mTOR, Akt and P70S6K in thymic epithelial cells derived from seven patients with A, AB or B thymomas. Interestingly, all the primary cells expressed detectable levels of phosphorylated Akt and mTOR while phospho-P70S6K was low or barely detectable (Fig 4). This suggests that the activation of the Akt/ mTOR pathway might participate to the cell proliferation associated with tumor growth.

### Effects of rapamycin treatment on tumoral TECs proliferation

**Fig 5:**
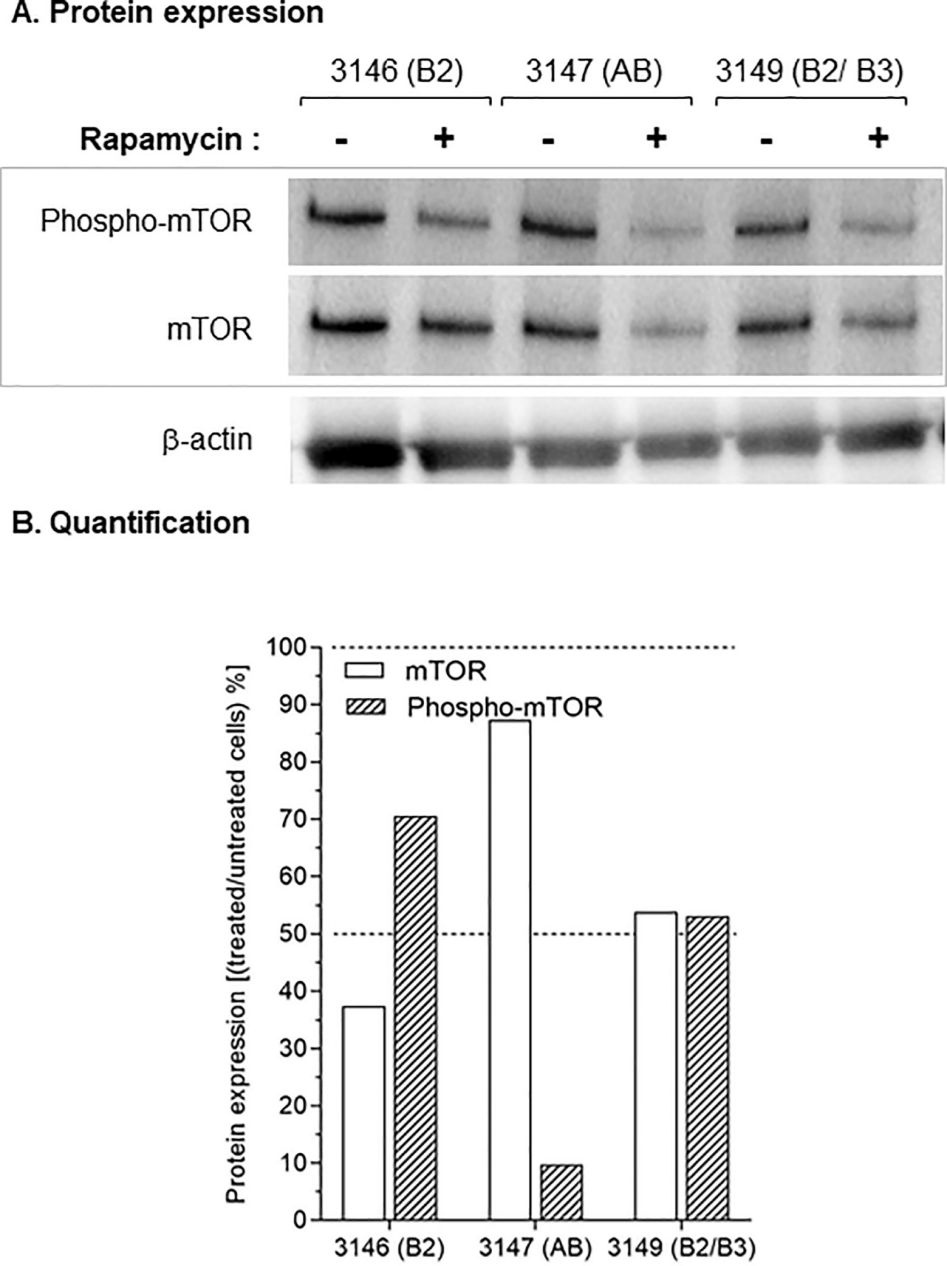
Inhibition of mTOR and phospho-mTOR expression upon rapamycin treatment of thymoma- derived cells. A. Expression of mTOR and phospho-mTOR proteins in thymoma-derived cells after 48 hours treatment with 100 nM rapamycin (+) or no rapamycin (-). B. Protein expression of mTOR (with bars) and Phospho-mTOR (hatched bars) has been measured and expressed as the ratio of protein expression in [treated/ untreated] cells.

We analyzed the effect of rapamycin, an inhibitor of mTOR, on proliferation of primary thymic epithelial cells. We focused our analysis on cell cultures derived from AB (# 3147), B2 (# 3146) and B2/ B3 (#3149) thymomas at early passages because these cells had good proliferative abilities (~24 hours doubling time), a prerequisite for the study of rapamycin inhibition over a 72-hour period. Importantly, these three thymomas and the derived thymic epithelial cells expressed mTOR and phospho-mTOR (Figs 3 and 4). We controlled the inhibition of mTOR and phospho-mTOR in cells treated for 24 hours with 100 nM rapamycin. The activation of mTOR was reduced by ~90%, ~30% and ~ 50% in thymic epithelial cells respectively derived from AB (3147), B2 (3146) and B2/B3 thymomas (Fig 5).

**Fig 6:**
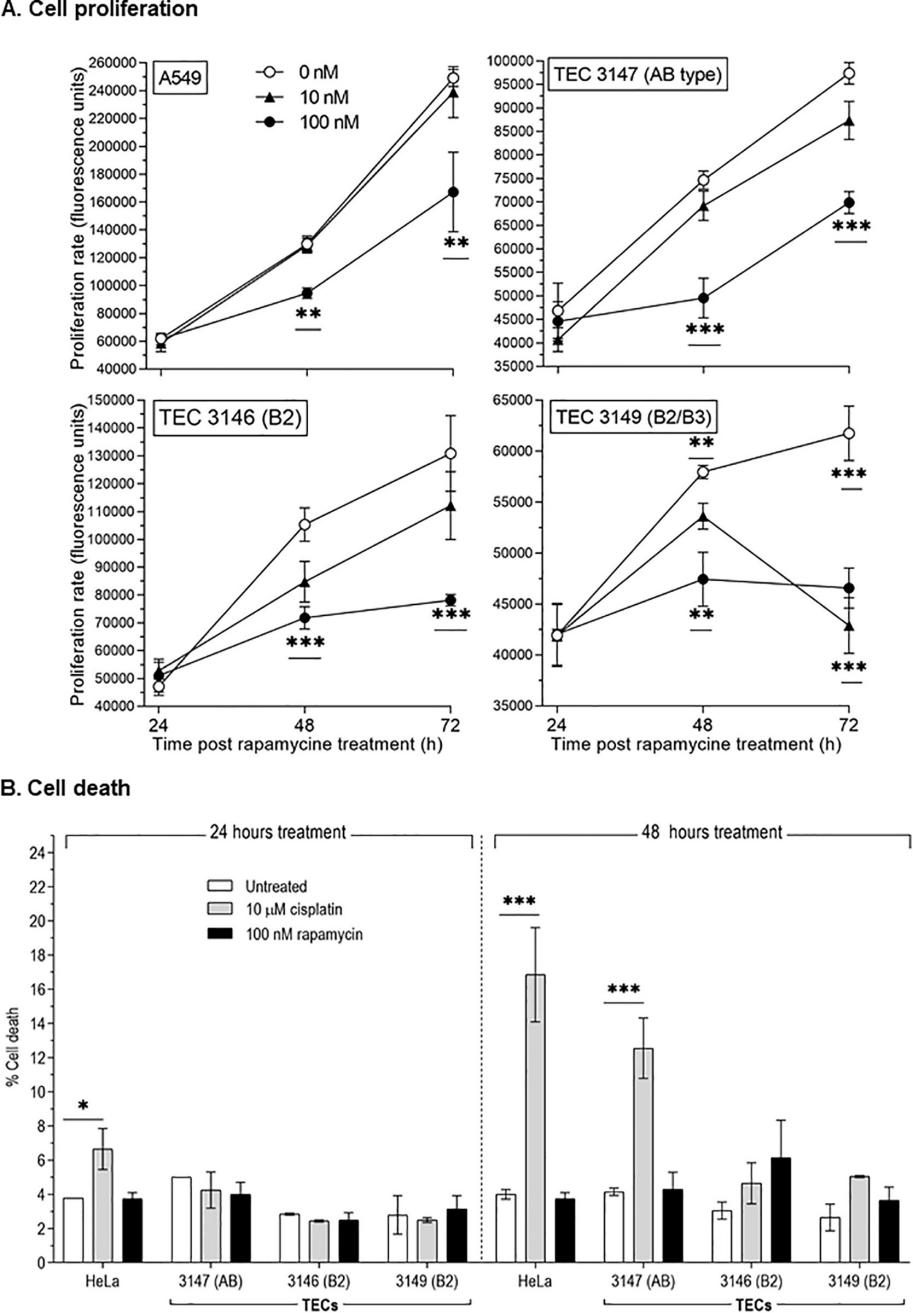
Proliferation and cell death of TECs upon rapamycin treatment. 1. Proliferation rate has been measured in TECs derived from AB (#3147), B2 (#3146) and B2/ B3 (#3149) thymomas after 24, 48 and 72 hours of culture with 0 nM (○), 10 nM (▲) or 100 nM (●) rapamycin. 2. Cell death was measured by flow cytometry and expressed as percentage of cell death in TECs treated for 24 or 48 hours with 100 nM rapamycin (′) or 10 μM cisplatin (′). Statistical significance (α = 0.05) with t test; ** <0.02; *** < 0.001.

The proliferation rate was measured daily, in triplicates and repeated at least twice over 72-hour treatment with 1 nM, 10 nM or 100 nM rapamycin. No effect was detectable with 1 nM rapamycin; while with 10 or 100 nM, the proliferation was significantly reduced in all three primary cell cultures (Fig 5). When compared to untreated cells, 100 nM rapamycin significantly blocked cell proliferation with a 40%, 25% and 29% decrease of the cell number for TECs derived respectively from thymomas 3146, 3149 and 3147. The inhibitory effect of rapamycin was similar to what we observed (~30%) in A549 cells treated with 100 nM rapamycin (Fig 6A).

We measured cell death using flow cytometry in TECs treated for 24 and 48 hours with 100 nM rapamycin or 10 μM cisplatin. Rapamycin had no significant effect on cell death. Interestingly, only TECs 3147 derived from a B2 lymphoma significantly died upon cisplatin treatment (Fig 6B).

## Discussion

Deciphering the molecular events in thymomas has remained a major challenge given the rarity and the histological heterogeneity of those tumors, precluding large genomic studies to be conducted. The presence of lymphocytes intermixed with epithelial tumoral cells in tissue may lead to potential misinterpretation of genomic features specifically associated with thymic carcinogenesis [27, 30, 31]. In this study, we report the derivation of primary thymic epithelial cell cultures from type A, AB and B thymomas, their phenotypic and genetic characterization as well as the deregulation of the Akt-mTOR pathway and its impact on cell proliferation.

Previous reports of derivation of cells from thymic epithelial tumors have been made available. Most cell lines were obtained from thymic carcinoma specimens, with limited molecular characterization. Besides PIK3 regulatory subunits mutations [24], copy number gain of the anti-apoptotic molecule BCL2 was observed at comparative genomic hybridization of such cell lines, while *in vitro* siRNA knockdown reduced cell proliferation, and *in vivo* exposure to a pan-BCL2 inhibitor led to an inhibition of xenograft growth, *via* a mechanism involving the PIK3/AKT/mTOR pathway [23]. Exposure of thymic carcinoma cells to HSP90 inhibitors led to cell cycle arrest and apoptosis, and blocked invasiveness, through the downregulation of HSP90 oncogenic clients, including insulin-like growth factor 1 receptor (IGF-1R), a transmembrane tyrosine kinase receptor frequently overexpressed in thymic carcinomas, CDK4, and PIK3/ Akt [32]. Taken together, these data were of significant therapeutic relevance: while pictilisib is mostly developed in breast cancers, which more frequently harbor PIK3 alterations, phase II trials dedicated to thymic epithelial tumors were conducted with the IGF-1R inhibitor cixutumumab [33], the mTOR inhibitor everolimus [34], and the CDK inhibitor milciclib [INS], reporting on clinical antitumor activity in advanced, refractory cases. Meanwhile, the IU-TAB-1- cell line was established from type AB thymoma, with phenotypic and molecular profiling but limited information of derivation protocol and success rate, and subsequent analysis of molecular pathways of interest, including PIK3/ AKT/ mTOR [35].

In our study, we were able to derive primary thymic epithelial cells from all twelve patients immediately after tumor removal, and we have successfully maintained and expanded the cells *in vitro*. We provide the community with a reliable protocol that worked not only for thymic carcinomas but also for thymomas which are known to have lower proliferation index associated with slow growth and better outcome.

We used primary thymic epithelia cells to study the deregulation of the Akt-mTOR signaling pathway and the efficacy of rapamycin to block cell proliferation, thus providing with a helpful tool to validate findings from high-throughput analysis on thymic tissues. We demonstrated that the Akt-mTOR pathway was activated in thymomas as well as in thymic epithelial tumor cells derived from type A, AB, and B thymomas. The proliferation of these cells was significantly reduced after exposure to rapamycin through the decrease of mTOR phosphorylation, in absence of significative cell death. The Akt/ mTOR pathway might be an important player for the tumor development and a good target for drugs in patients. Rapamycin specifically inhibits mTORC1, and many reports highlight the role of mTORC2 in cancer [36]. From our data, phosphorylation of mTORC1 target p70S6K together with the AKT phosphorylation at Ser473, would support the potential activation of both mTORC1 and mTORC2 in thymomas. Ultimately, our findings showing activation of the Akt-mTOR pathway in thymomas are of significant clinical relevance, given the recent results of a phase II study of everolimus in advanced thymic epithelial tumors, reporting on a disease control rate of 88%, with median progression-free survival of 10.1 months and median overall survival of 25.7 months [34]. Everolimus is currently available and may represent an off-label option for refractory tumors [3]. In the future, more specific inhibitors of the PIK3/ AKT/ mTOR pathway may be evaluated in those tumors.

Beside the deregulation of the Akt/ mTOR pathway, we have identified for the first time PIK3CA mutation in a type B2/B3 thymoma, which may participate to the deregulation of the Akt-mTOR pathway, among others [37]. PIK3 activation was also reported to be related to overexpression of a microRNA cluster on chr19q13.42 in type A and AB thymomas, observed in IU-TAB1 cell line [38]. This alteration was observed in the cohort of The Cancer Genome Atlas [39]. Interestingly, only one of our cases harbored a GTF2I mutations, that was associated at RPPA analysis in this cohort, with lower expression of the apoptosis, cell cycle, DNA damage response, hormone receptor signaling, breast hormone signaling, RAS/MAPK, RTK, and TSC/mTOR pathways [39].

In conclusion, our data enhance the potential role of thymic epithelial cells derived from tissue specimens for an *in vitro* exploration of molecular abnormalities specific to thymic carcinogenesis. This may be relevant in a research setting to assess the value of molecular alterations observed at high-throughput genomic profiling, and develop in vivo models, but also to develop approaches for precision medicine strategies at the patient individual level.
